# Effect of Ultrasonic Nanocrystal Surface Modification on the Microstructure and Martensitic Transformation of Selective Laser Melted Nitinol

**DOI:** 10.3390/ma12193068

**Published:** 2019-09-20

**Authors:** C.A. Biffi, P. Bassani, M. Nematollahi, N. Shayesteh Moghaddam, A. Amerinatanzi, M.J. Mahtabi, M. Elahinia, A. Tuissi

**Affiliations:** 1 National Research Council; Institute of Condensed Matter Chemistry and Technologies for Energy, CNR ICMATE, Unit of Lecco, Via Previati 1E, 23900 Lecco, Italy; paola.bassani@cnr.it (P.B.); ausonio.tuissi@cnr.it (A.T.); 2 Dynamic and Smart Systems Laboratory, Mechanical Industrial and Manufacturing Engineering Department, The University of Toledo, Toledo, OH 43606, USA; m.nematolahi@gmail.com (M.N.); mohammad.elahinia@utoledo.edu (M.E.); 3 Mechanical and Aerospace Engineering, University of Texas at Arlington, Arlington, TX 76019, USA; narges.shayestehmoghaddam@rockets.utoledo.edu (N.S.M.); amirhesam.amerinatanzi@rockets.utoledo.edu (A.A.); 4 Department of Mechanical Engineering, University of Tennessee at Chattanooga, Chattanooga, TN 37403, USA; mohammad.javad.mahtabioghani@utoledo.edu

**Keywords:** NiTi, selective laser melting, ultrasonic nano-crystal surface modification, EBSD, microstructure, XRD

## Abstract

Nitinol has significant potential for biomedical and actuating-sensing devices, thanks to its functional properties. The use of selective laser melting (SLM) with Nitinol powder can promote novel applications aimed to produce 3D complex parts with integrated functional performances. As the final step of the production route, finishing processing needs to be investigated both for the optimization of the surface morphology and the limit alteration of the Nitinol functional properties. In this work, the effect of an advanced method of surface modification, ultrasonic nanocrystal surface modification (UNSM), on the martensitic transformation and microstructure of SLM built Ni50.8Ti49.2 (at.%) was investigated. Scanning electron microscopy, X-ray diffraction, and differential scanning calorimetry indicated that the UNSM process can generate stress-induced martensite, at least partially suppressing the martensitic transformation. The microhardness profile indicates that the UNSM process can affect the mechanical properties of the SLMed Nitinol sample in a range of up to approximately 750 μm in depth from the upper surface, while electron backscatter diffraction analysis highlighted that the initial austenitic phase was modified within a depth below 200 μm from the UNSMed surface.

## 1. Introduction

Quasi-equiatomic NiTi alloys are the most diffused shape memory alloys (SMAs), thanks to their stable and optimal functional properties, namely shape memory effect and pseudoelasticity [1]. These properties depend on martensitic transformation (MT) and can be found in Ti-rich NiTi and Ni-rich NiTi alloys above or below room temperature, respectively. As the operating temperatures of the MT are affected strongly by the Ni/Ti ratio, the manufacturing of the NiTi SMAs is very challenging for getting stable functional characteristics [2].

In recent years, the use of additive manufacturing (AM) techniques for realizing 3D parts in NiTi alloys has become an attractive solution for advanced devices [3,4,5,6,7]. The feasibility of manufacturing Nitinol through different AM technologies, like selective laser melting (SLM), electron beam melting, and direct energy deposition, has been demonstrated in several experimental works [8,9,10]. Of the AM methods, the SLM process is the most investigated one, since it can offer the best compromise between product quality, productivity, and diffusion in the market. The MT and the related functional properties of SLM-built Nitinol parts have been shown to be almost comparable to those of the wrought alloy under compression testing [11,12,13], while the tensile behavior is less consolidated, due to the problem of residual porosity [14,15]. The compositional issue remains an aspect to be well controlled during the laser process in order to allow an appropriate Ni/Ti ratio for inducing the MT [16,17]. Moreover, the microstructure obtained by SLM is strongly textured and this can be optimized for enhancing the functional response of the NiTi alloy [18].

However, this is not enough for fixing the final performance of the device. In detail, SLM often requires the adoption of post-processing treatments, not only for inducing tailored microstructures through heat treatments but also surface modifications [19,20], due to high roughness values and relevant dimensional errors [21,22]. The literature reports some works regarding the performances of conventional treatments, like electropolishing [23,24], and advanced finishing post-processing, like laser shock peening [25,26] and ultrasonic nanocrystal surface modification (UNSM) [27,28,29], on NiTinol samples. The main achievements obtained during the surface modification processing were good surface roughness values and improved surface responses, such as wear resistance, fatigue behavior, and biological response.

So far, only a few of these works have focused on SLMed NiTi parts [24,29]. As the initial surface condition and the mechanical properties of a rolled NiTi tape or wire are pretty different from the ones of an SLMed component, the performances of the post-processing treatments should be revised for the AMed NiTi alloys. In fact, in a previous work the effect of the main SLM process parameters on the performances of electropolishing on NiTi samples containing interior channels was studied [24]. It was found that electropolishing can decrease the roughness only for low power densities, thanks to the smaller melt pools and columnar structures. In another work, the effect of the UNSM treatment on the surface morphology, mechanical and wear properties, as well as the corrosion resistance was studied [29]. The principal results were that a deformed structure can be induced, provoking an increase in the hardness in correspondence with the post-processed surface. Additionally, the surface quality was improved and a reduction in the residual porosity was achieved. However, no effect of this innovative post-processing method was investigated on the functional properties of NiTi SLMed parts. For this reason, the present work has the goal of investigating the evolution of the microstructure, which is correlated to the MT, of superelastic NiTi produced by the SLM process.

## 2. Experimental

**Figure 1:**
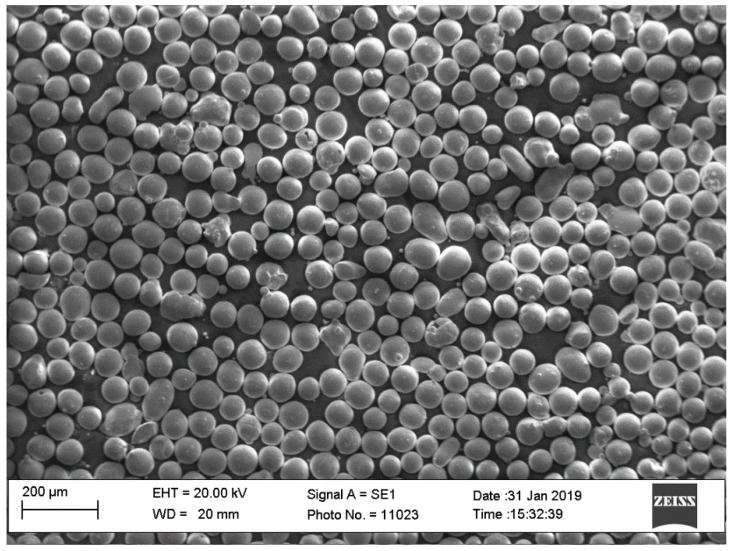
Scanning electron microscope image of the Nitinol powders used for the realization of the selective laser melting (SLM)-built samples.

The as-cast Ni_50.8_Ti_49.2_ (at.%) ingot (Nitinol Devices & Components, Inc., Fremont, USA) was gas atomized by TLS Technique GmbH to produce powder, as shown in Figure 1, characterized by a size distribution in the range 25–75 μm. An SLM system (mod. PXM from Phenix/3D Systems) was used for manufacturing cylindrical samples (10 mm in diameter and 2 mm in height); the axis of the cylindrical sample was placed parallel to the SLM building direction, indicated as the z axis. The process conditions, used for the manufacturing of the samples, are reported in Table 1.

The UNSM process was performed using a tungsten carbide tip (2.4 mm in diameter) in oscillation at 20 kHz [12]. First, a static load of 3 kg was applied, while the oscillation was characterized by an amplitude of 10 microns and a scanning speed of 2 m/min. The surface modification treatment was performed on the upper surface of the SLMed cylinder, lying on the x–y plane and perpendicular to the building direction (z axis). Further details of the UNSM process were reported elsewhere [29].

The surface roughness, profile, and mapping were realized by a Keyence VHX-6000 series digital microscope. Then, the samples were cut in order to analyze both the x–y and x–z plane orientations. First, x–y surfaces were prepared without mounting. Polishing steps were performed, paying attention to remove as little as possible material from the x–y surfaces. The x–z section was embedded in conductive graphite-loaded mounting resin, and prepared with conventional metallographic polishing techniques (grinding with emery papers and final polishing with diamond suspensions). An additional chemomechanical polishing step was performed with colloidal silica to allow electron back scattered diffraction (EBSD) analyses.

The microstructures of as-built and UNSMed specimens were evaluated through light optical microscopy (OM, mod. Leitz Aristomet) and scanning electron microscopy (W-SEM Leo1430, Zeiss, and FEG-SEM SU70, Hitachi). The texture of the specimens was investigated through electron backscatter diffraction (EBSD), performed at different magnifications, with 20 kV acceleration voltage and step size in the range 0.5–2 µm. The mechanical properties of the samples were evaluated by Vickers microhardness profiles, performed by using a Leitz Miniload hardness tester, applying a 100 g load for 10 s. Differential scanning calorimetry (DSC) tests were carried out on specimens (30 mg weight) cut from the full section, which included the modified surface layer and untreated base material. DSC analyses were performed using a Seiko DSC220C with a heating/cooling rate of 10 °C/min within a temperature range of −100 °C to 100 °C; three complete cycles were performed for each sample. X-ray diffraction patterns were collected by a diffractometer (Panalytical X’Pert Pro) using Cu K_α_ radiation, operating at 40 kV and 30 mA in the 20°–100° 2theta range. The patterns were acquired at temperatures of −60 °C and 60 °C, temperatures selected after DSC results in order to ensure complete phase transformation into martensite or austenite, respectively.

## 3. Results and Discussion

**Figure 2:**
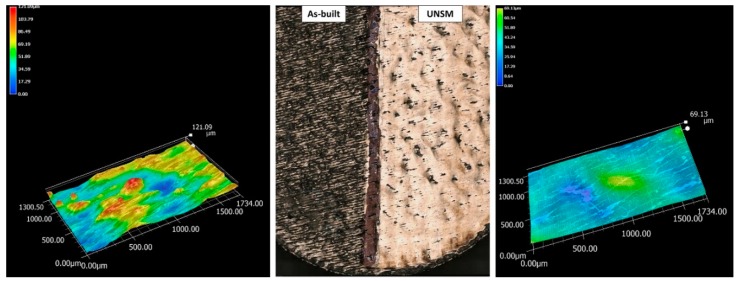
Macrograph of the sample, showing on the left the as built surface and on the right the UNSMed surface with the corresponding 3D profilometry measurements.

Figure 2 shows the macrograph of the sample, partially post-processed and partially left in the as-built condition, and the corresponding 3D profilometric measurements. The as-built surface indicates the presence of irregularities, depending on the balling effect, due to the melting of the powder and followed by solidification. Preferential direction can be seen, which depends on the scanning direction adopted during the last processed layer. Evidence of residual traces associated with the laser scanning lines on the UNSMed surface can be found, but the plastic deformation process limited the difference between peaks and valleys in the roughness profile, according to the color bar. Moreover, the roughness values were not only reduced but also evened out, to the 3D roughness profiles, and the average roughness varied from 12 µm down to 9 µm through the UNSM processing.

**Figure 3:**
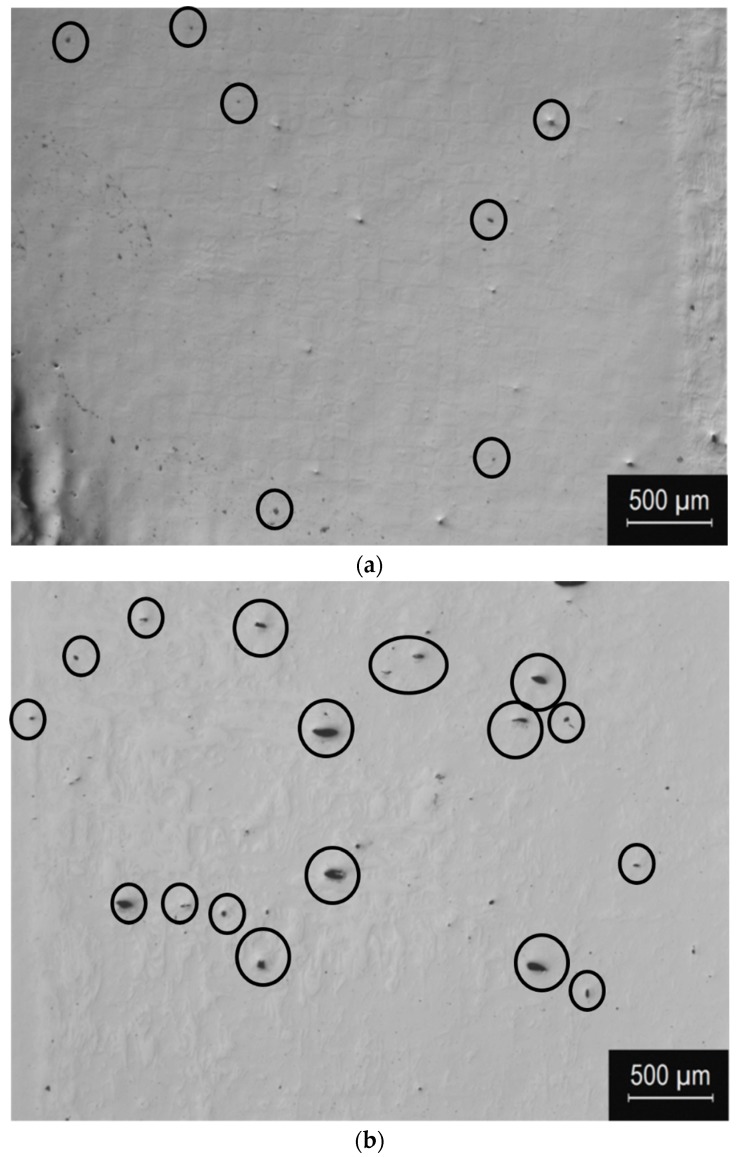

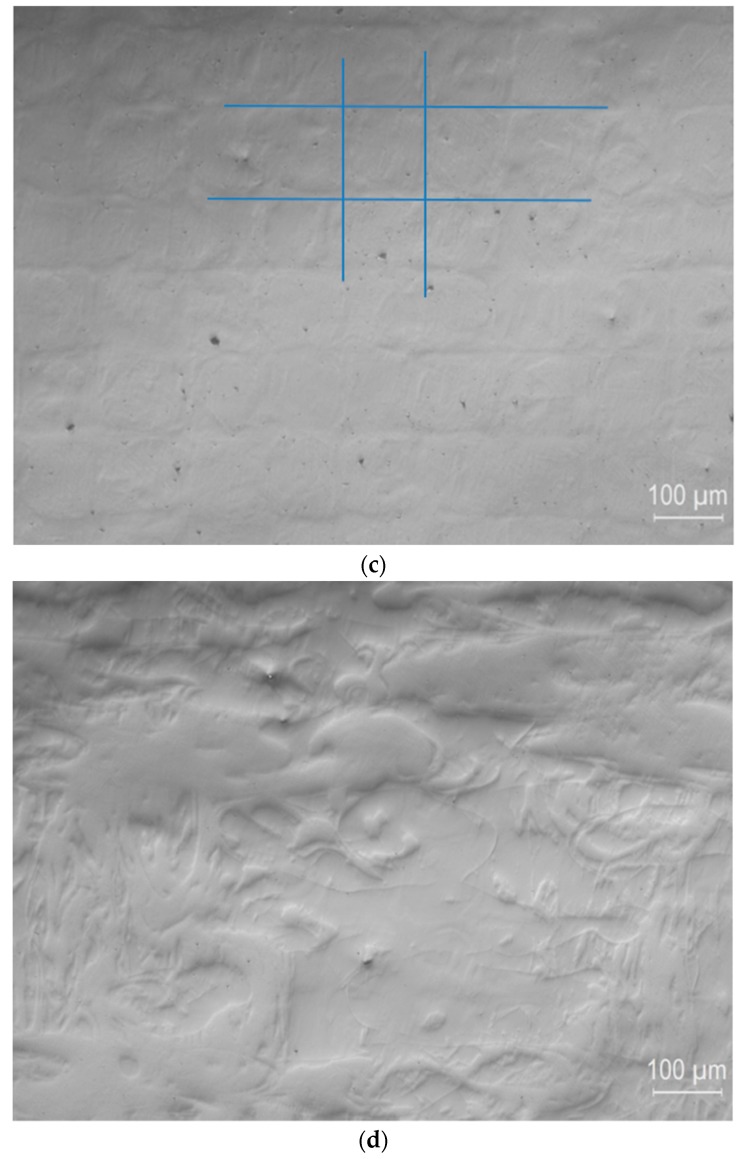
Micrographs showing planar x–y views of the UNSMed (**a**–**c**) and the as-built surfaces (**b**–**d**), acquired by optical microscope at two magnifications.

In Figure 3, micrographs at two magnifications, acquired with OM, are presented of the UNSMed and as-built surfaces. Low magnification micrographs are representative of the porosities present just near the upper surface of the samples. A comparison of Figure 3a,b reports the partial occlusion of the pores, placed in the x–y view, due to the plastic deformation induced by the UNSM process [29]. In particular, the post-processing can significantly reduce the pore size, as can be seen from the porosity evolution highlighted from the circles in Figure 3a,b. At higher magnifications, the variation in the microstructure can be observed. Figure 3d shows the microstructure of the as-built material: the traces of the liquid pools provoked by laser along the scanning direction are clearly recognizable, and are characterized by bright features at melt pool boundaries. After the UNSM process, a squared pattern (see blue colored lines in Figure 3c), of approximately 120 µm edge size, can be appreciated, which mimics the texture of the material due to the 90° building strategy. This effect is due to local plastic deformation, which mitigates typical features of solidification melt pools (faint segregations at melt pools boundaries), and enhances underlying texture effects. Further analysis at higher magnifications, performed by SEM, can better clarify the microstructural modifications.

**Figure 4:**
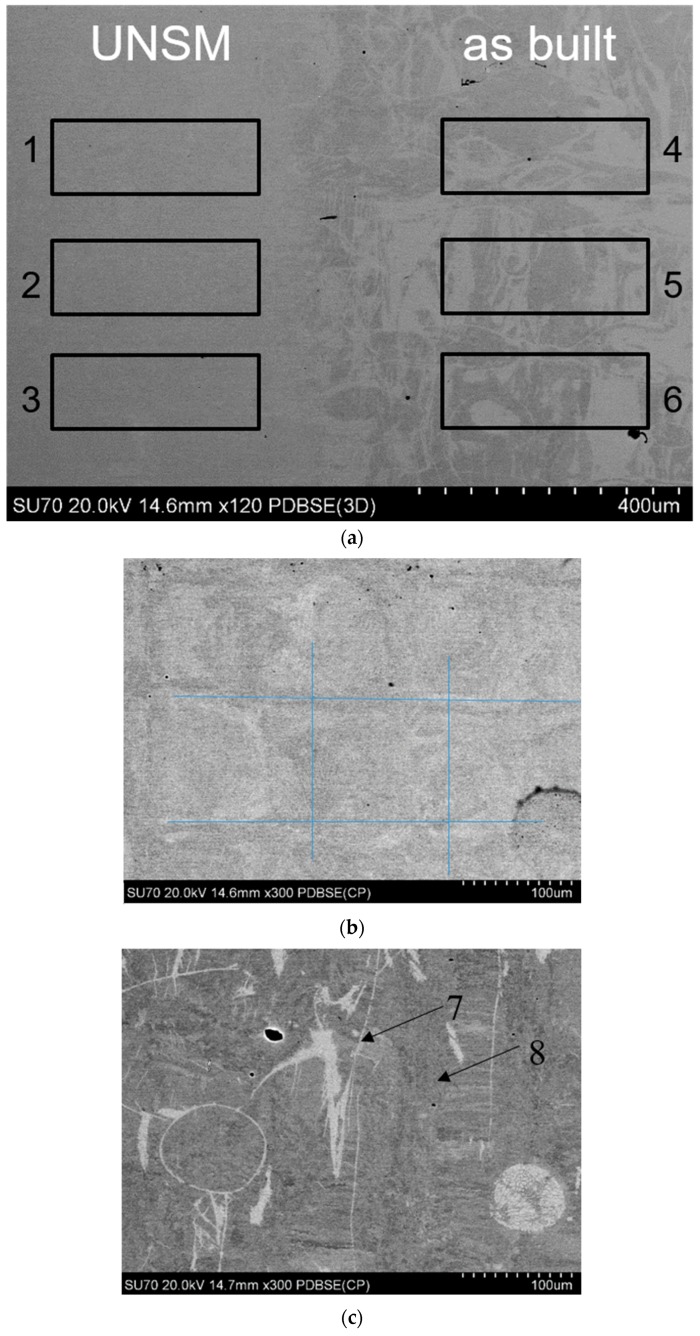
BSE-SEM micrographs in the x–y view: surface containing the border between depicting the UNSMed and the initial surfaces (**a**); higher magnification of the UNSMed (**b**) and as-built surface (**c**).

Figure 4 shows the SEM micrographs acquired using backscattered electrons (BSE) in the planar x–y view of the UNSMed and as-built surfaces. Figure 4a reports the surfaces, before (right side) and after (left side) the UNSM process: backscatter contrast is more pronounced in the as-built side, while the UNSM side shows far less evident contrast. Three regions for each condition were analyzed by energy dispersive spectroscopy (EDS) and no compositional difference was observed. Elemental analysis is reported in Table 2. As expected, the plastic deformation did not provoke any loss of Ni or Ti elements or oxidation. Moreover, the variability in the composition was slightly limited and an equiatomic Ni:Ti ratio was maintained. Figure 4b,c shows the surfaces at higher magnification. The UNSMed surface is characterized by the squared footprints, previously observed in Figure 2d, with relatively low contrast. On the contrary, the SEM image of the as-built surface indicates the presence of areas with different BSE contrast, evidencing the presence of microstructural features correlated to the melt pool morphology and to incomplete melting of some powders, too. The strong contrast of these areas was mainly due to microstructural differences. EDS measurements were performed on the light and dark areas, respectively indicated as point 7 and 8 in Table 3, and the resulting Ni:Ti ratios do not indicate any significant compositional modification.

**Figure 5:**
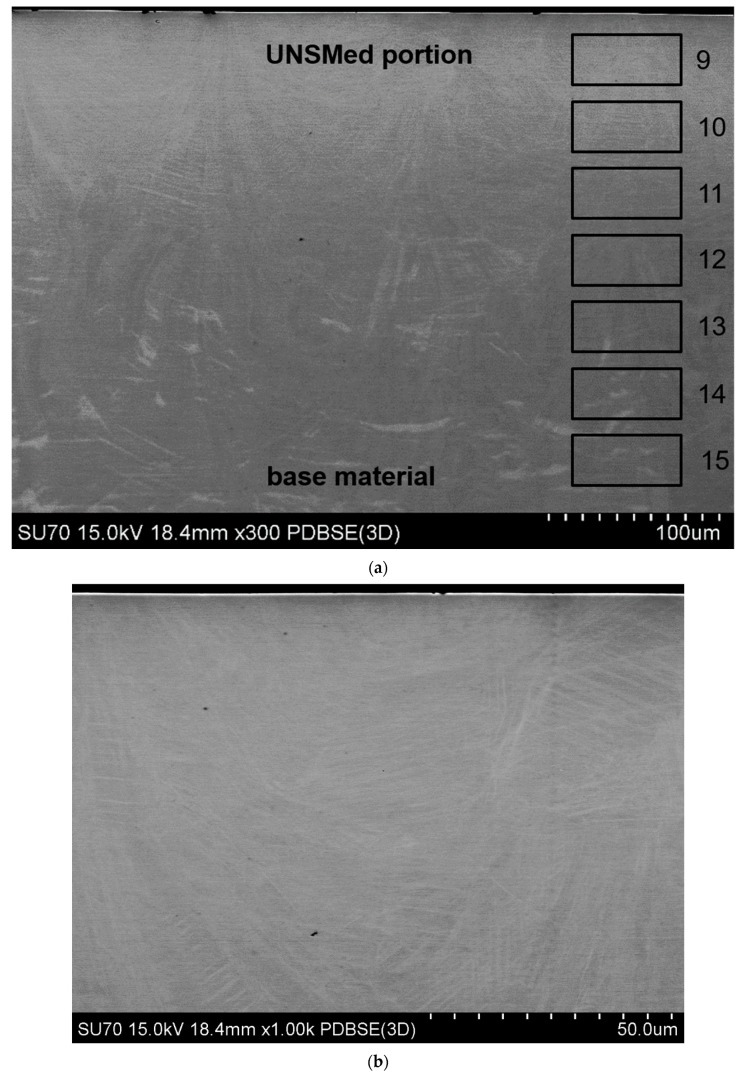

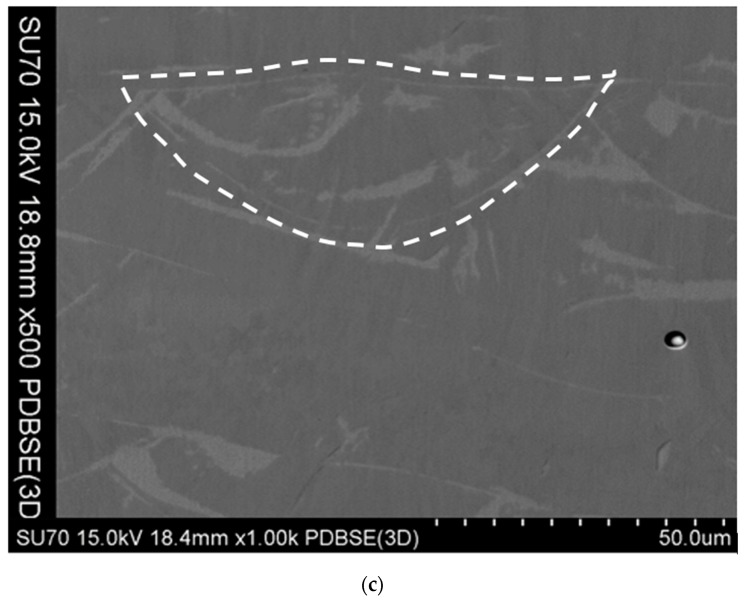
Back scattered electron (BSE)-SEM image in the x–z view of the UNSMed sample (**a**), showing the regions for the EDS compositional profile; magnifications of the UNSMed portion (**b**) and the base material, with an highlighted melt pool boundary (**c**).

BSE-SEM observations were also performed along the building direction, indicated as the x–z view. In Figure 5a, the x–z view of the UNSMed sample is shown. It can be seen that the upper portion of the NiTi sample is characterized by a different microstructure with respect to the underlying material, representing both as-built and UNSMed material. A compositional profile was performed (see the region from 9 to 15 of Figure 4a) and the corresponding results are listed in Table 3. As expected, no compositional modification was observed along the building direction. On the contrary, the plastic work of the USNM process induced clear modification of the microstructure of the SLMed NiTi alloy. Figure 5b,c depicts the microstructure obtained by the surface modification process and a reference one. The typical microstructure of AMed parts, in which small overlapping liquid pools are due to the layer by layer building strategy, cannot be seen in the UNSMed portion. In fact, plastic deformation and amorphization [28] induced by UNSM hindered the BSE texture sensitivity; moreover, lamellar features, grouped in colonies of similar orientation, can be observed, possibly deformation bands or stress-induced martensite. Moving far from the upper surface, the as-built material is characterized by a microstructure similar to that reported in Figure 3c, in which the presence of the adjacent liquid pools, due to the laser traces, and related inhomogeneities in BSE contrast can be detected. No other differences were observed between the as-built and UNSMed material.

**Figure 6:**
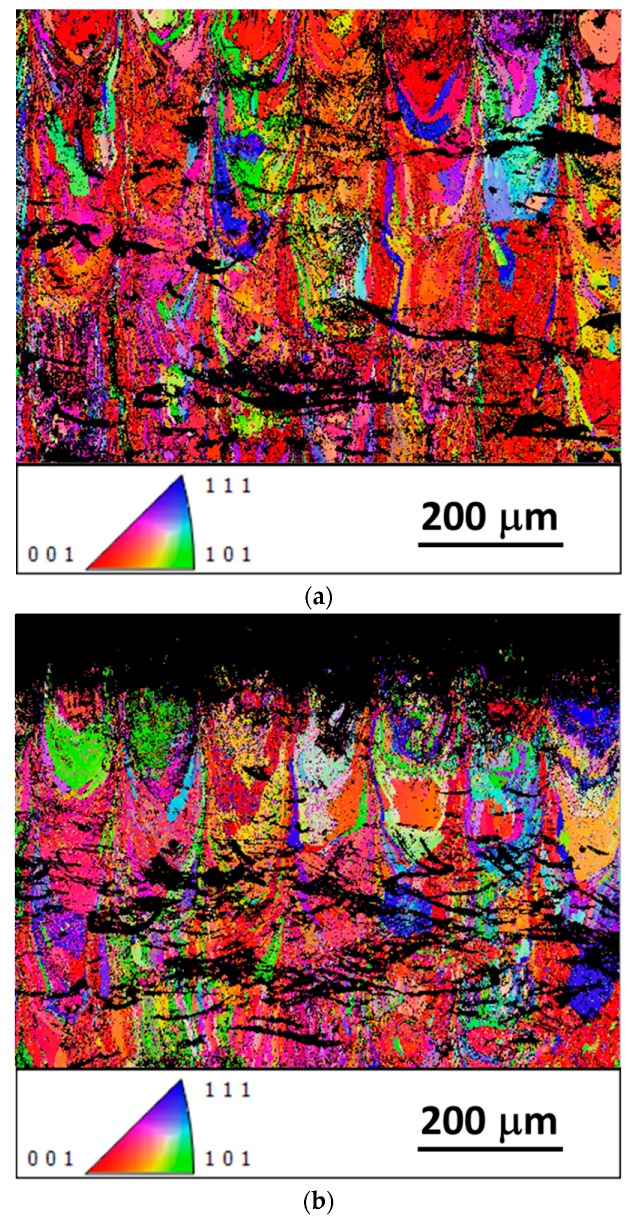
Electron backscatter diffraction (EBSD) analysis performed in the x–z view of the two conditions: as-built (**a**) and UNSMed samples (**b**), according to orientation map micrograph related to the building direction.

**Figure 7:**
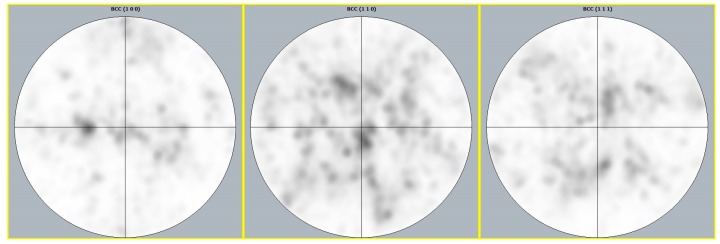
Pole figures of the as-built sample.

Figure 6 shows the EBSD analysis performed on the x–z view for both of the conditions. After the rapid solidification characteristic of the SLM process, the structure contained elongated grains, orientated along the building direction (see Figure 6a). The material was characterized by a weak texture, with (001) aligned with the building direction. The UNSM process can cause severe plastic deformation to the surface layers, leading to lattice distortion and crystal defects, which significantly weaken Kikuchi patterns. Consequently, a wide non-indexed area appeared in the treated region, as shown in Figure 6b. Additional unsolved pixels inside as-built material seem to be related to locally stress-induced martensite (SIM) or to martensite induced by small chemical composition variations. The local plastic deformation can be estimated at a depth below 200 µm. In Figure 7, pole figures of the as-built sample are shown; no intense preferential texture can be observed. This can be associated with the selected process condition not able to induce strong preferential crystal orientations during the SLM process [11,18].

**Figure 8:**
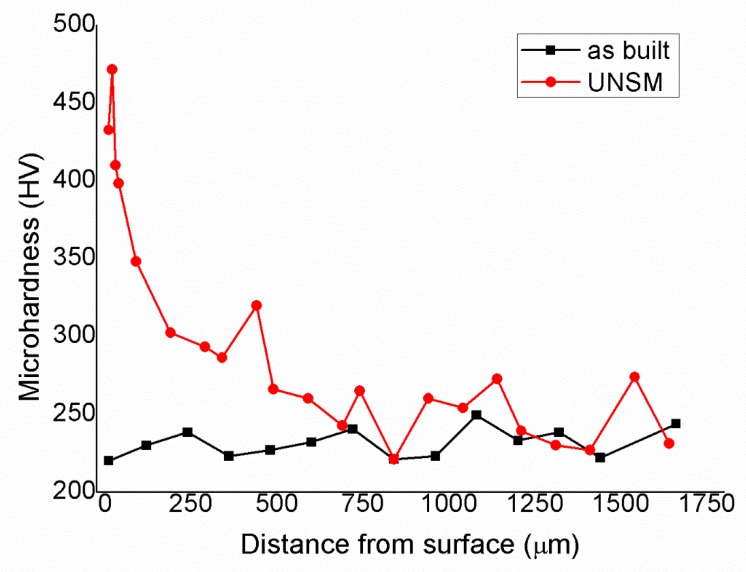
Microhardness profiles of the as-built and UNSMed samples.

Due to the microstructure modification induced by the UNSM process, a sensible variation in mechanical behavior can be expected. The evolution of the microhardness is plotted in Figure 8. The UNSM-treated sample exhibited an increase in microhardness values on the upper surface up to 470 HV, which can be attributed to the plastic deformation induced by the postprocessing. The effect of the UNSM process disappeared at a penetration of approximately 750 µm from the surface, reaching around 240 HV in microhardness in the as-built material.

This increase in hardness could depend on high stress values generated during the UNSM process, able to provoke relevant plastic deformation and therefore induce cold working. The stress applied during the UNSM process is higher than the stress required for mechanically inducing the martensite (usually in the range of 400–500 MPa), because it is able to induce partial cold working on the upper surface of the NiTi sample.

The MT of the samples in both of the conditions, as-built and UNSMed, was investigated by coupling DSC and XRD analyses. According to Figure 8, there was not a noticeable difference in MT behavior between the DSC samples. This is quite reasonable, as the USNM process does not influence a thick portion of the NiTi sample but it is confined in a limited depth [29], as shown by Figure 8. The MT is stable upon three complete thermal cycles and the alloy is fully superelastic at body temperature. Table 4 reports the values of the transformation temperatures and the transformation enthalpies, measured from the DSC scans on the third cycle. Due to the unclear ending point of direct transformation, values of Mf and transformation enthalpies are affected by measurement uncertainty. Nethertheless, the transformation temperatures did not seem to be influenced by the sample condition. The observed temperatures shift was very limited and did not suggest any significant alterations in the material properties.

Moreover, the transformation enthalpies remained rather constant in the range 18–19 J/g after the USNM process, which is in good agreement with the NiTi reference [1]. This indicates that the modification was confined only to the machined surface and the massive NiTi did not suffer any significant modifications, not only in the chemical composition but also in the microstructure, which has high sensibility to plastic deformation [30].

**Figure 9:**
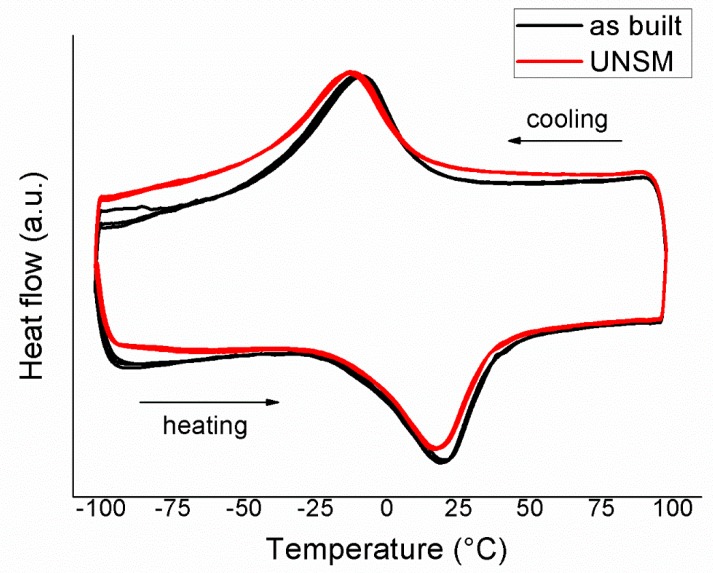
DSC scans of the as-built and UNSMed samples.

**Figure 10:**
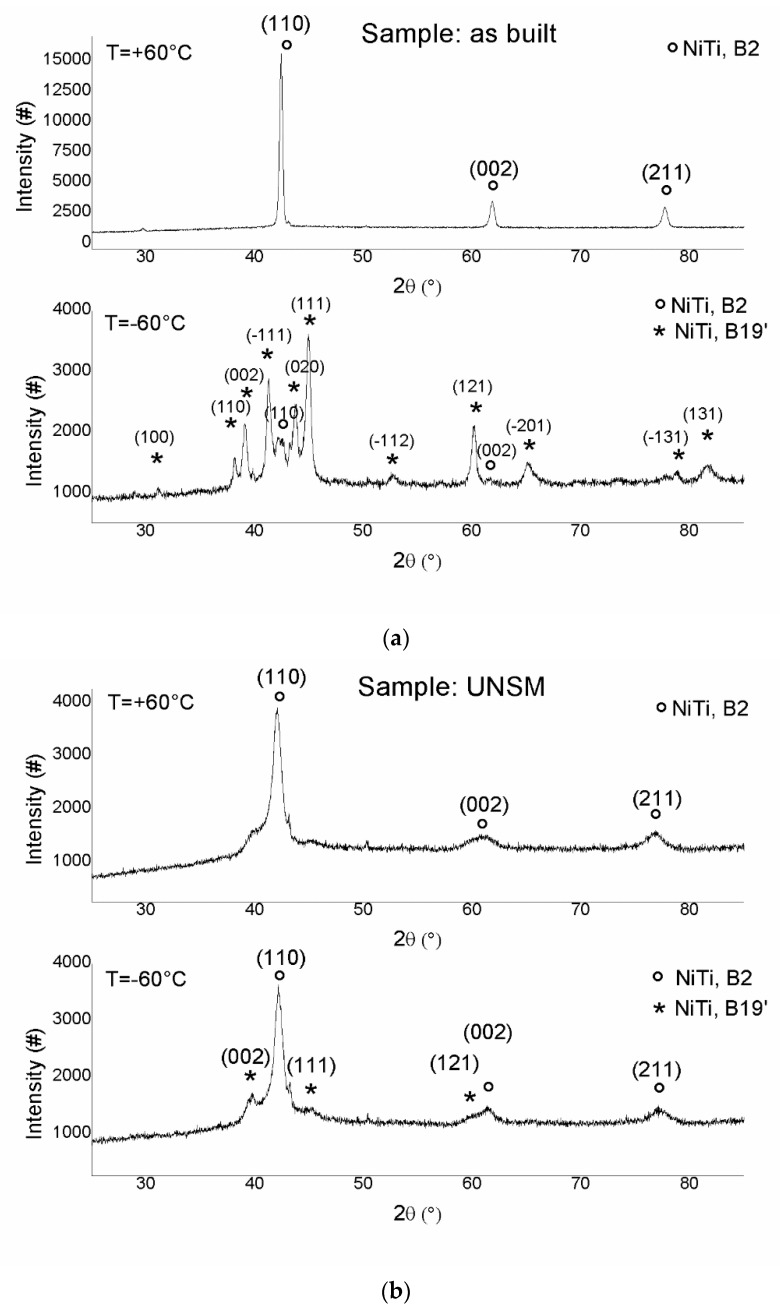
XRD patterns at two temperatures (−60 °C and 60 °C) of as-built (**a**) and UNSMed (**b**) surfaces.

Figure 10a,b depict the XRD spectra, performed at temperatures of −60 °C and 60 °C, of as-built and UNSMed surfaces, respectively. The temperatures were selected in order to control the evolution of the microstructure after the complete MT from austenite (+60 °C) into martensite (−60 °C). The as-built sample showed a complete MT; a B2 austenite phase was identified at high temperature, while the B19′ structure was detected at low temperature. No evident residual austenite was found at −60 °C in the as-built sample. This analysis is in good agreement with the DSC investigation of Figure 9b.

On the contrary, the USNM-treated sample showed a different behaviour: at high temperature, the sample had a B2 austenitic structure, according to the DSC measurement. The main difference with the as-built sample is that the B2 peaks were far broader, indicating a highly defective austenitic structure and possibly also stress-induced martensite in the microstructure. The relative intensities of the (110), (002), and (211) austenitic peaks were similar in the two conditions, indicating that no abrupt change in the texture occurred. Few modifications in the microstructure were detected upon temperature varyiation, and consisted in a slight increase of the humps in the martensite peak angular regions. This means that the plastic deformation induced by the USNM process can induce a cold working sufficient to mostly suppress the MT in the first few microns from the upper surface. However, the degree of plastic deformation associated with the UNSM process performed on the investigated sample induced a broadening effect of the B2 peak (110), as shown in Figure 10b, but no complete surface amorphization, as reported in the literature [28]. This is an important result, because it can be stated that the UNSM process can be designed for the occlusion of the pores, improving the surface quality and limiting the lattice distortion for minimizing the cristallinity alteration.

## 4. Conclusions

The effect of an advanced finishing process, namely, ultrasonic nano-crystal surface modification, on the microstructure and martensitic transformation of an SLMed Nitinol sample was investigated. The following conclusions can be highlighted:

(1) The UNSM process can offer benefits in the post-processing of AM products, being able to occlude surface pores and slightly decrease the roughness;
(2) Due to the mechanical energy deposited on the Nitinol sample, the texture induced by the SLM process varied after the UNSM treatment without any compositional variation;
(3) The local plastic deformation suppressed the martensitic transformation at a depth estimated to be below 200 µm by EBSD analysis, due to the plastic deformation induced by the post-processing;
(4) The microhardness profile indicated a deeper variation in the mechanical properties, down to 750 µm;
(5) In this work, the microstructure obtained on the upper surface after the post-processing contained both austenite and martensite, but NiTi amorphization was avoided. This is a different result from literature. As a consequence, the selection of the main parameters of the ultrasonic nano- changes;
(6) The improvement of the mechanical properties of the surface could enhance the fatigue behavior of the post-processed parts.

## Figures and Tables

**Table 1:** Process parameters used for the manufacturing of the Nitinol samples.

Laser Power	Layer Thickness	Scanning Speed	Hatch Distance	Laser Beam Size
250 W	30 µm	1250 mm/s	120 µm	80 µm

**Table 2:** Quantitative compositional results from EDS measurements, performed in the planar x–y view. The indication regarding the localization of the region of measurement is indicated in Figure 4a,c. For all measurements, an average 0.3% sigma was obtained.

Sites	Ti Content (at. %)	Ni Content (at. %)
1	49.88	50.12
2	50.25	49.75
3	49.97	50.03
4	50.01	49.99
5	50.07	49.93
6	49.66	50.34
7	49.76	50.24
8	49.40	50.60

**Table 3:** Compositional results from EDS measurements, performed in the x–z view. The indication regarding the localization of the region of measurement is indicated in Figure 5a. For all measurements average 0.3% sigma was obtained.

Sites	Ti Content (at. %)	Ni Content (at. %)
9	48.84	51.16
10	49.21	50.79
11	49.41	50.79
12	48.89	51.11
13	49.57	50.43
14	49.12	50.88
15	49.06	50.94

**Table 4:** Transformation temperatures and enthalpies during the MT of as-built and UNSMed samples.

Samples	H_M__→A _(J/g)	As (°C)	Ap (°C)	Af (°C)	H_A__→M _(J/g)	Ms (°C)	Mp (°C)	Mf (°C)
As-built	18.7	−18	20	40	19.5	14	−8	−46
UNSM	19	−16	18	39	17.9	13	−11	−40
